# An Investigation of the RNA Modification m^6^A and Its Regulatory Enzymes in Rat Brains Affected by Chronic Morphine Treatment and Withdrawal

**DOI:** 10.3390/ijms26094371

**Published:** 2025-05-04

**Authors:** Anna Hronova, Eliska Pritulova, Lucie Hejnova, Jiri Novotny

**Affiliations:** Department of Physiology, Faculty of Science, Charles University, 128 00 Prague, Czech Republic; anna.hronova@natur.cuni.cz (A.H.); eliska.pritulova@natur.cuni.cz (E.P.); lucie.hejnova@natur.cuni.cz (L.H.)

**Keywords:** FTO, N6-methyladenosine, morphine, morphine withdrawal, rat brain

## Abstract

N6-methyladenosine (m^6^A) is one of the most prevalent methylated modifications of mRNA in eukaryotes. This reversible alteration can directly or indirectly influence biological functions, including RNA degradation, translation, and splicing. This study investigates the impact of chronic morphine administration and varying withdrawal durations (1 day, 1 week, 4 weeks, and 12 weeks) on the m^6^A modification levels in brain regions critical to addiction development and persistence. Our findings indicate that in the prefrontal cortex, the m^6^A levels and METTL3 expression decrease, accompanied by an increase in FTO and ALKBH5 expression, followed by fluctuating, but statistically insignificant changes in methylation-regulating enzymes over prolonged withdrawal. In the striatum, reductions in m^6^A levels and METTL3 expression are observed at 4 weeks of withdrawal, preceded by non-significant fluctuations in enzyme expression and the m^6^A modification levels. In contrast, no changes in the m^6^A modification levels or the expression of related enzymes are detected in the hippocampus and the cerebellum. Our data suggest that m^6^A modification and its regulatory enzymes undergo region-specific and time-dependent changes in response to chronic morphine exposure and subsequent withdrawal.

## 1. Introduction

The N6-methyladenosine (m^6^A) modification is one of the most abundant and evolutionarily conserved post-transcriptional modifications, present in all organisms, ranging from yeast to mammals [1]. The methylation of the amino group at the N6 position of adenine bases is a dynamic and reversible process mediated by the interplay of enzymes: writers, erasers, and readers [2]. m^6^A plays a crucial role in post-transcriptional regulation by influencing pre-mRNA splicing, nuclear export, stabilization, degradation, and translation [3,4]. The methylation of adenosine at the N6 position is catalyzed by a methyltransferase multiprotein complex, which includes METTL3 (methyltransferase-like 3), METTL14 (methyltransferase-like 14), WTAP (Wilms tumor 1-associated protein), VIRMA (vir-like m^6^A methyltransferase-associated protein), METTL16 (methyltransferase-like 16), RBM15, and ZC3H13 (zinc finger CCCH domain-containing protein 13) [5,6,7,8,9,10,11]. METTL3 serves as the catalytic subunit, while the other enzymes in the complex contribute to complex stabilization, target RNA recognition, proper nuclear localization, and synergistically enhance METTL3’s catalytic activity [12]. The methyltransferase complex transfers a methyl group from the substrate S-adenosyl methionine (SAM) to the adenine bases in acceptor RNA substrates [13]. The removal of m^6^A marks is mediated by “eraser” enzymes, including FTO (fat mass and obesity-associated protein) and ALKBH5 (AlkB homolog 5), both of which belong to the AlkB homolog family of iron(II)- and α-ketoglutarate-dependent dioxygenases [14]. “Reader” proteins recognize m^6^A modifications and influence the RNA fate by regulating stability, translation, transport, and degradation. These proteins contain YTH domains and are classified into three groups: YTHDC1, YTHDC2, and the YTHDF family (YTHDF1, YTHDF2, and YTHDF3). The YTHDF proteins, localized in the cytoplasm, primarily facilitate the translation and degradation of m^6^A-modified mRNA [15,16,17]. In contrast, YTHDC1 and YTHDC2 are predominantly nuclear, where they regulate mRNA splicing, translation, and degradation [18,19,20,21].

The m^6^A modification plays a crucial role in brain development, learning and memory, synaptic plasticity, stress responses, and the regulation of mood disorders [22,23,24,25,26,27]. Additionally, m^6^A modifications are known to be key regulators in the development of various cancers [28,29,30,31,32,33]. Although research on the relationship between m^6^A modifications and addiction remains limited, some studies have begun to explore this connection. For example, in cocaine addiction, the downregulation of FTO and increased m^6^A levels have been observed in the hippocampus [34]. In contrast, other studies on acute and chronic morphine exposure in mice found no significant impact on m^6^A modifications in the brain [35]. However, another study investigating chronic morphine administration in primary cortical cultures reported an increase in m^6^A modifications [36]. These findings highlight the need for further research into the role of m^6^A RNA modification in addiction, particularly in comparison to other epigenetic mechanisms that have been more extensively studied in the context of opioid dependence. For instance, changes in histone methylation, including H3K9me2 and H3K4me2, have been linked to the increased expression of the transcription factor ∆FosB, a key regulator of addiction development [37,38,39]. Long-term opioid use also induces significant alterations in DNA methylation, with both increases and decreases in 5-methylcytosine (5mC) and 5-hydroxymethylcytosine (5hmC) levels across various brain regions [40,41]. These epigenetic modifications underscore the profound and lasting impact of chronic opioid use on neurobiology, which may persist even after opioid consumption has ceased. Given the intricate role of m^6^A modifications and other epigenetic mechanisms in neural function and gene regulation, further investigation is essential to understand how substances with addictive potential influence these processes.

Morphine, a potent opioid widely used in clinical practice for its analgesic and anxiolytic properties, not only modulates the central nervous system to relieve pain, but also interacts with neural pathways that may lead to long-term changes in gene expression [42,43,44,45]. The epigenetic modifications induced by morphine highlight its dual role as both a crucial therapeutic agent and a substance with high addictive potential [46,47]. Understanding the interplay between morphine and epigenetic mechanisms, such as m^6^A RNA modifications, is essential for elucidating the molecular basis of addiction and its long-term consequences.

Significant gaps remain in our understanding of the spatiotemporal dynamics of epigenetic regulation in the brain during withdrawal and relapse. In this study, we examined m^6^A modification levels and the expression of key enzymes (METTL3, ALKBH5, FTO, and YTHDF1) across four brain regions—the prefrontal cortex, hippocampus, cerebellum, and striatum—at four withdrawal time points, extending up to 12 weeks after morphine cessation.

## 2. Results

### 2.1. Assessment of m^6^A Levels and Key RNA Methylation/Demethylation Enzymes in the Prefrontal Cortex

**Figure 1:**
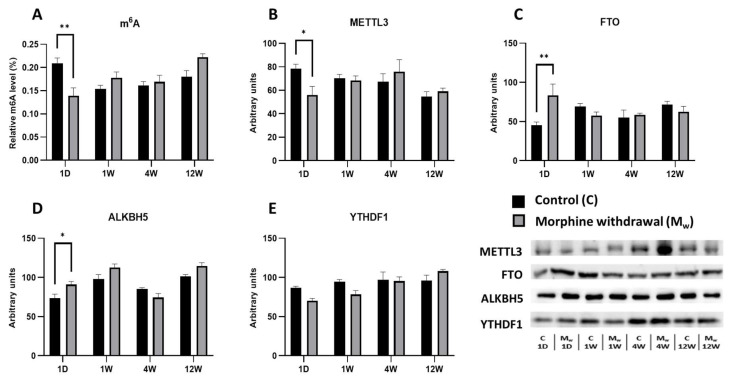
The determination of N6-methyladenosine, METTL3, FTO, ALKBH5, and YTHDF1 levels in the prefrontal cortex. The proportion of m^6^A modification was assessed and expressed as a percentage of total RNA methylation (**A**). The Western blot analysis of METTL3 (**B**), FTO (**C**), ALKBH5 (**D**), and YTHDF1 (**E**) was performed as described in the Section 4. The relative protein levels were quantified via the densitometric analysis of the corresponding bands on the Western blots and normalized to the appropriate internal standard (set at 100%). The data were obtained from five biological replicates (*n* = 5), and statistical significance was assessed using two-way ANOVA, followed by the Bonferroni post-hoc test. The bars represent the mean ± SEM (* *p* ≤ 0.05, ** *p* ≤ 0.01).

**Figure 2:**
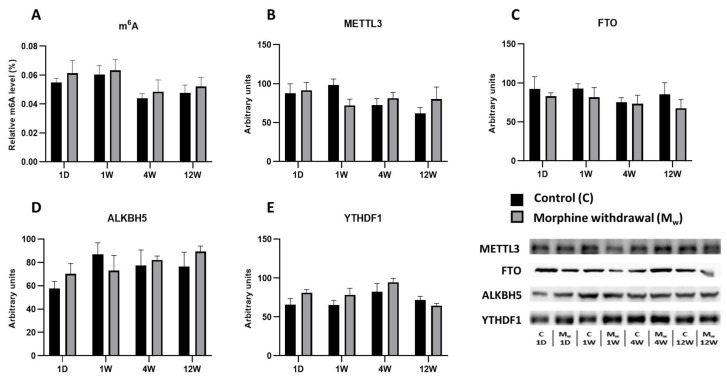
The determination of N6-methyladenosine, METTL3, FTO, ALKBH5, and YTHDF1 levels in the hippocampus. The proportion of m^6^A modification was assessed and expressed as a percentage of total RNA methylation (**A**). The Western blot analysis of METTL3 (**B**), FTO (**C**), ALKBH5 (**D**), and YTHDF1 (**E**) was performed as described in the Methods section. The relative protein levels were quantified via the densitometric analysis of the corresponding bands on the Western blots and normalized to the appropriate internal standard (set at 100%). The data were obtained from five biological replicates (*n* = 5), and statistical significance was assessed using two-way ANOVA, followed by the Bonferroni post-hoc test. The bars represent the mean ± SEM.

The chronic administration of morphine at a dose of 10–50 mg/kg/day for 10 days, followed by withdrawal, led to changes in the prefrontal cortex, affecting both the m^6^A levels and the key enzymes involved in RNA methylation and demethylation, including METTL3, FTO, ALKBH5, and the m^6^A-reading protein YTHDF1 (Figure 1). In the morphine-treated group that abstained for 1 day, the m^6^A levels were significantly decreased compared to those of the control group (1D: *p* = 0.0017, Figure 2A). This reduction in m^6^A was accompanied by a significant decrease in METTL3 expression (1D: *p* = 0.0455, Figure 1B), the key enzyme responsible for catalyzing m^6^A modification, suggesting that METTL3 suppression contributed to the observed decline in m^6^A levels. Similarly, the level of FTO, a demethylase that removes m^6^A modifications, was significantly increased in the morphine-treated group at this time point (1D: *p* = 0.0055, Figure 1C). In contrast, ALKBH5, another demethylase, showed a significant decrease in expression in the morphine-treated group (1D: *p* = 0.0313, Figure 1D). At the later withdrawal time points (1 week, 4 weeks, and 12 weeks), the m^6^A levels exhibited a tendency to increase in the morphine-treated group, but did not reach statistical significance (1W, 4W, and 12W: *p* > 0.05). Similarly, METTL3 expression showed no significant differences at these time points (1W, 4W, and 12W: *p* > 0.05). The FTO and ALKBH5 levels also fluctuated, but did not differ significantly from the control group after longer withdrawal periods (1W, 4W, and 12W: *p* > 0.05 for both the enzymes). In contrast, the expression of YTHDF1, the protein that recognizes and binds to m^6^A modifications, remained unchanged in the morphine-treated groups compared to the respective controls (1W, 4W, and 12W: *p* > 0.05, Figure 1E).

### 2.2. Assessment of m^6^A Levels and Key RNA Methylation/Demethylation Enzymes in the Hippocampus

In the same model of morphine administration followed by withdrawal, no changes in the m^6^A modification levels were observed in the hippocampus (Figure 2). At no time point during the withdrawal period was a significant difference detected between the control and morphine-treated groups (1W, 4W, and 12W: *p* > 0.05, Figure 2A). Similarly, the expression of the methyltransferase METTL3 showed no statistically significant differences between the groups at any time point. The largest difference was observed after one week of withdrawal; however, this difference was not significant (1W: *p* = 0.2979, Figure 2B). The expression of the demethylases FTO and ALKBH5 (Figure 2C,D) fluctuated over time, but none of these changes were statistically significant. The ALKBH5 levels in the morphine-treated group tended to be higher than those in the control group after one day of withdrawal, though this difference was not significant (*p* > 0.05). Similarly, no significant changes were observed in the expression of the reader protein YTHDF1 at any time point (1W, 4W, and 12W: *p* > 0.05, Figure 2E).

### 2.3. Assessment of m^6^A Levels and Key RNA Methylation/Demethylation Enzymes in the Striatum

**Figure 3:**
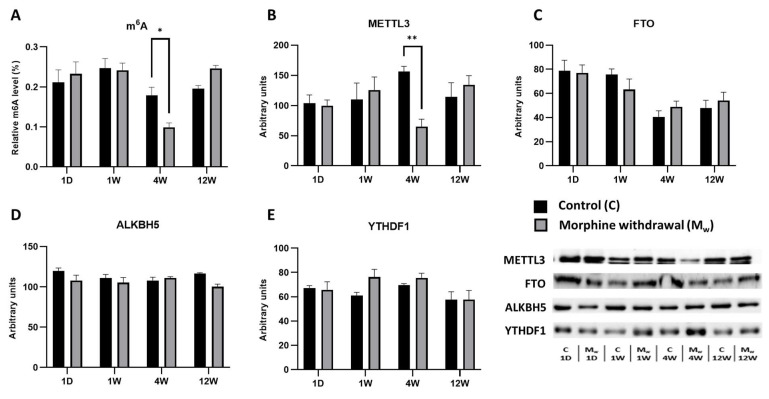
The determination of N6-methyladenosine, METTL3, FTO, ALKBH5, and YTHDF1 levels in the striatum. The proportion of m^6^A modification was assessed and expressed as a percentage of total RNA methylation (**A**). The Western blot analysis of METTL3 (**B**), FTO (**C**), ALKBH5 (**D**), and YTHDF1 (**E**) was performed as described in the Methods section. The relative protein levels were quantified via the densitometric analysis of the corresponding bands on Western blots and normalized to the appropriate internal standard (set at 100%). The data were obtained from five biological replicates (*n* = 5), and statistical significance was assessed using two-way ANOVA, followed by the Bonferroni post-hoc test. The bars represent the mean ± SEM (* *p* ≤ 0.05, ** *p* ≤ 0.01).

**Figure 4:**
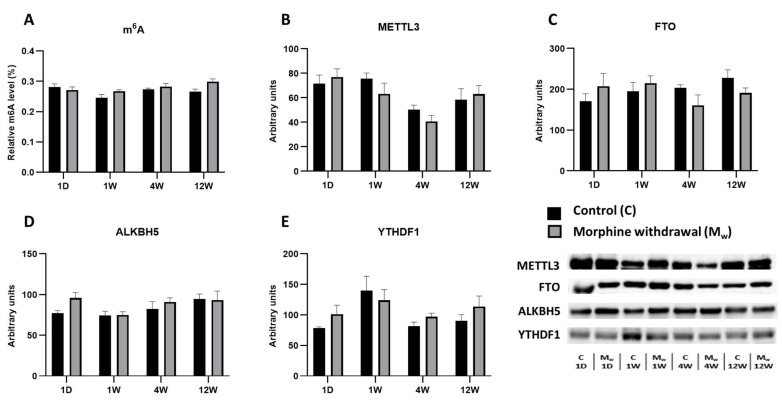
The determination of N6-methyladenosine, METTL3, FTO, ALKBH5, and YTHDF1 levels in the cerebellum. The proportion of m^6^A modification was assessed and expressed as a percentage of total RNA methylation (**A**). The Western blot analysis of METTL3 (**B**), FTO (**C**), ALKBH5 (**D**), and YTHDF1 (**E**) was performed as described in the Methods section. The relative protein levels were quantified via the densitometric analysis of the corresponding bands on the Western blots and normalized to the appropriate internal standard (set at 100%). The data were obtained from five biological replicates (*n* = 5), and statistical significance was assessed using two-way ANOVA, followed by the Bonferroni post-hoc test. The bars represent the mean ± SEM.

In the striatum, the alterations in the m^6^A levels and the enzymes regulating this modification became evident only after an extended period of withdrawal (Figure 3). In the morphine-treated group, no statistically significant changes were observed after 1 day or 1 week of withdrawal (*p* > 0.05, Figure 3A). However, after 4 weeks of withdrawal, a more significant decrease in m^6^A levels was detected in the morphine-treated group compared to that of the control group (4W: *p* = 0.0448, Figure 3A). By contrast, at 12 weeks of withdrawal, no statistically significant difference was observed (12W: *p* = 0.3798, Figure 4A). The decrease in m^6^A levels at 4 weeks of withdrawal in the morphine-treated group was accompanied by the significant upregulation of the methyltransferase METTL3 (4W: *p* = 0.0072, Figure 3B). At the other withdrawal time points, METTL3 expression exhibited fluctuations, but did not reach statistical significance (1D, 1W, and 12W: *p* > 0.05, Figure 3B). The expression of FTO, a demethylase that removes m^6^A modifications, did not show statistically significant changes between the control and morphine-treated groups at any time point (1D, 1W, 4W, and 12W: *p* > 0.05, Figure 4C). Similarly, the expression of ALKBH5, another demethylase, remained stable across all the time points, with no significant differences between then groups (1D, 1W, 4W, and 12W: *p* > 0.05, Figure 3D). Unlike the enzymes involved in m^6^A modification, the expression of YTHDF1, a protein that recognizes and binds to m^6^A modifications, did not significantly differ between the control and morphine-treated groups at any time point (1D, 1W, 4W, and 12W: *p* > 0.05, Figure 3E).

### 2.4. Assessment of m^6^A Levels and Key RNA Methylation/Demethylation Enzymes in the Cerebellum

In this brain region, we analyzed the m^6^A modification levels along with the enzymes involved in its regulation (METTL3, FTO, ALKBH5, and YTHDF1). As with the other brain structures, we compared the expression levels of these enzymes and m^6^A modifications between the control and morphine-treated groups at various time points (1D, 1W, 4W, and 12W) (Figure 4). In the morphine-treated group, the m^6^A modification levels did not significantly differ from those of the control group at any time point (1D, 1W, 4W, and 12W: *p* > 0.5, Figure 4A). Similarly, the levels of the methyltransferase METTL3 showed no significant differences between the groups (1D, 1W, 4W, and 12W: *p* > 0.05, Figure 4B). The expression of the demethylase FTO also showed no statistically significant changes between the control and morphine-treated groups. While some fluctuations were observed, none reached statistical significance (1D, 1W, 4W, and 12W: *p* > 0.05, Figure 4C). Similarly, the levels of ALKBH5, another demethylase, remained stable, with no significant differences between the groups at any time point (1D, 1W, 4W, and 12W: *p* > 0.05, Figure 4D). Similar to the other enzymes, the expression of the reader protein YTHDF1 did not show any statistically significant changes at any time point (1D, 1W, 4W, and 12W: *p* > 0.05, Figure 4E).

## 3. Discussion

The aim of this study was to investigate the effects of chronic morphine administration and subsequent withdrawal on m^6^A RNA modification and the associated regulatory enzymes in rat brains. The experiments were conducted on adult male Wistar rats, which received intraperitoneal morphine in escalating doses (10–50 mg/kg/day) over a period of 10 days. This model was selected based on previous research demonstrating its ability to induce dependence and tolerance [48]. The withdrawal periods lasted 1 day, 1 week, 4 weeks, and 12 weeks.

While epigenetic modifications such as histone and DNA methylation have been extensively studied in the context of opioid dependence [37,38,39,40,41], RNA modifications—particularly m^6^A—remain underexplored. m^6^A modification is one of the most abundant modifications and is dynamically regulated by a network of enzymes. These include (1) the methyltransferase complex (comprising METTL3, METTL14, METTL16, WTAP, VIRMA, RBM15, and ZC3H13), which catalyzes m^6^A addition; (2) the demethylases FTO and ALKBH5, which remove m^6^A modifications; and (3) the reader proteins YTHDF1/2/3 and YTHDC1/2, which interpret the modification’s effects. This study focused on four key enzymes: METTL3, FTO, ALKBH5, and YTHDF1. METTL3, the catalytic component of the methyltransferase complex, transfers a methyl group from S-adenosylmethionine (SAM) to adenine in RNA [12,13]. FTO and ALKBH5 are demethylases from the AlkB homolog family that remove m^6^A modifications [14]. YTHDF1, a predominantly cytosolic reader protein, facilitates the translation of m^6^A-modified mRNA and may contribute to its degradation [16]. The recent findings suggest that YTHDF1 may also modulate neuroinflammatory pathways during chronic morphine exposure, thereby contributing to the development of morphine tolerance and hyperalgesia [49]. To assess the impact of chronic morphine exposure on the expression of these enzymes, we performed protein isolation and Western blot analysis. Additionally, RNA was extracted, and the m^6^A levels were quantified using a commercial ELISA kit. This integrated approach enabled the correlation of enzyme expression patterns with m^6^A modifications across distinct brain regions. Our research focused on four key brain structures—the prefrontal cortex, the hippocampus, the striatum, and the cerebellum—due to their critical roles in neurobiological processes underlying addiction.

In the prefrontal cortex, a key region of the reward system involved in opioid use, a more significant reduction in N6-methyladenosine (m^6^A) levels was observed in the morphine-treated group on the first day of withdrawal compared to that of the controls. This reduction was accompanied by the decreased expression of the METTL3 methyltransferase and the increased expression of the demethylases ALKBH5 and FTO. In the striatum (STR), a central region in addiction processes, a significant decline in the m^6^A modification levels was detected after 4 weeks of withdrawal, corresponding with decreased METTL3 expression. In contrast to the PFC and STR, no significant changes in the m^6^A modification levels or the expression of its regulatory enzymes were observed in the hippocampus (HIP) or the cerebellum (CRB). These findings suggest that the effects of morphine withdrawal on m^6^A modification are highly region-specific and may occur through indirect mechanisms.

Chronic morphine administration, followed by overnight withdrawal, led to significant changes in the prefrontal cortex, particularly in the m^6^A levels and the expression of the enzymes regulating this modification. One day of withdrawal—a period characterized by strong withdrawal symptoms, such as heightened anxiety and motor restlessness [50]—was associated with a significant decrease in the m^6^A levels. This decrease was accompanied by the reduced expression of the METTL3 enzyme and the increased expression of the demethylases FTO and ALKBH5. These findings suggest that rapid RNA demethylation occurs during the early withdrawal phase in response to morphine cessation. By one week of withdrawal, these effects were no longer observed, indicating that the time interval may have been too long for significant changes to persist. Similarly, at the later withdrawal stages (4 and 12 weeks), no significant alterations in the m^6^A levels or enzyme expression were detected. This indicates that the observed changes are likely a short-term, localized response to acute withdrawal, potentially reflecting the gradual stabilization of the prefrontal cortex after morphine cessation.

A study using primary cortical cultures reported the opposite trend during chronic morphine administration, showing increased m^6^A levels and decreased ALKBH5 expression [36]. However, these differences may stem from the contrast between in vitro and in vivo conditions. While in vitro models allow for the detailed analysis of cellular mechanisms, they lack the complex regulatory networks present in the brain, which involve interactions between different regions and systemic responses to drug exposure. Consequently, the in vitro increase in m^6^A levels during chronic morphine exposure may reflect mechanisms specific to certain subpopulations of brain cells, whereas in vivo m^6^A regulation may be influenced by broader neuroadaptive processes linked to both drug exposure and withdrawal [51]. Further evidence for dynamic epitranscriptomic changes during withdrawal is provided by a recent study [52], which reported an increase in the methylation of GlyGCC C39 tRNA in the medial prefrontal cortex of mice after 30 days of morphine withdrawal. This increase was associated with the decreased expression of the corresponding tRNA, suggesting that long-term withdrawal may induce persistent epitranscriptomic adaptations even after acute m⁶A-related changes have subsided. Although this specific mechanism has not yet been described for m^6^A, it highlights the potential role of epitranscriptomic modifications in long-term neuroadaptations following opioid withdrawal.

In contrast, chronic morphine administration and subsequent withdrawal did not result in significant changes in the m^6^A levels in the hippocampus. At no point during the withdrawal period was a statistically significant difference observed between the control and morphine-treated groups. Similarly, the expression of the methyltransferase METTL3 remained unchanged, with the largest fluctuation occurring after 1 week of withdrawal, though this difference did not reach statistical significance. The expression of the demethylases FTO and ALKBH5 exhibited some variability over time, but none of these changes were statistically significant. Likewise, the levels of the reader protein YTHDF1 did not significantly differ between the groups, although a trend toward increased expression was observed in the morphine-treated group after 1 week of withdrawal, which did not reach statistical significance. These findings align with a recent study on mice, which also reported no significant changes in the hippocampal m^6^A levels or the expression of related enzymes following acute or chronic morphine administration. That study further demonstrated that the hippocampus was not activated after a single morphine exposure and that prolonged morphine administration did not induce notable alterations in m^6^A-related processes [35]. A possible explanation for these observations is that the hippocampus may not be primarily involved in the m^6^A-mediated responses to morphine exposure and withdrawal, unlike other brain regions such as the prefrontal cortex or the striatum. While morphine withdrawal led to decreased m^6^A levels and changes in METTL3 and demethylase expression in the prefrontal cortex and the striatum, the hippocampus remained resistant to these modifications. These findings support the hypothesis that the regulation of epigenetic modifications, including m^6^A methylation, varies across brain regions and that the hippocampus may not play a central role in these processes in the context of opioid exposure and withdrawal. Furthermore, the absence of significant changes in hippocampal m^6^A levels may be related to the distinct role of this brain region in addiction neurobiology. Unlike the prefrontal cortex and the striatum, which are directly involved in reward processing and drug-seeking behavior, the hippocampus primarily functions in memory and learning processes [24]. This suggests that hippocampal adaptations to morphine exposure may not be driven by m^6^A regulation, but rather by other molecular or structural mechanisms.

The m^6^A pathway plays a crucial role in synaptic plasticity and learning, underscoring its potential involvement in drug-induced neuroadaptation [25]. METTL3 and other components of the m^6^A machinery regulate the expression of genes associated with dopaminergic signaling, synaptic remodeling, and stress-related processes, mechanisms that are critically implicated in opioid addiction and withdrawal [26,53,54]. Emerging research suggests that opioid withdrawal induces epigenetic modifications that alter the gene expression in key addiction-related brain regions, including the striatum, the nucleus accumbens, and the prefrontal cortex [55,56,57,58]. Our findings indicate that morphine withdrawal leads to a significant, yet transient disruption of the m^6^A methylation pathway, characterized by a reduction in METTL3 expression and m^6^A levels at four weeks post-withdrawal. Given METTL3’s role in mRNA stability, translation, and synaptic plasticity, its downregulation in the striatum may contribute to the dysregulation of the dopaminergic system, an essential component in the development of opioid addiction and withdrawal syndrome [57,59,60]. The observed reduction in METTL3 aligns with the findings from other studies on stress-related and neurodegenerative disorders, suggesting a potential link between opioid withdrawal, stress resilience, and synaptic remodeling [61,62,63,64]. One possible mechanism contributing to these changes may involve alterations in glutamatergic neurotransmission. During the acute withdrawal phase, increased glutamatergic signaling has been reported in regions, such as the ventral tegmental area, the amygdala, and the nucleus accumbens, and is associated with symptoms such as anxiety and cravings [65]. Moreover, m^6^A modification has been shown to enhance the expression of the GluN2A subunit of NMDA receptors [66], suggesting that the early phase of withdrawal may be characterized by NMDA receptor overstimulation. The subsequent decrease in m^6^A levels and METTL3 expression observed after 4 weeks may therefore reflect a compensatory mechanism aimed at restoring synaptic balance following prolonged excitatory overstimulation. The 4-week withdrawal period may represent a critical window for neuroadaptations associated with opioid cessation. However, as these changes appear to be resolved by 12 weeks, our results suggest that such neuroepigenetic alterations are reversible rather than permanent [67,68].

While much of the existing research on opioid-induced neuroadaptations has focused on regions such as the prefrontal cortex, the striatum, and the hippocampus, comparatively less attention has been given to structures like the cerebellum. The cerebellum, a key center for motor coordination and learning, has not been extensively studied in the context of addiction and epigenetic modifications. However, the evidence suggests that opioids influence cerebellar function, affecting both neurotransmission and structural adaptations. Our results indicate that the m^6^A levels remained stable in the cerebellum throughout the withdrawal period, with no statistically significant changes observed at any time point. Similarly, the expression of the enzymes regulating m^6^A modification exhibited some fluctuations, but did not reach statistical significance. Likewise, the expression of the reader protein YTHDF1 did not show any significant changes at any time point, suggesting that opioid withdrawal does not affect m^6^A-mediated translational regulation in the cerebellum. These findings indicate that m^6^A modification and the enzymes involved in its regulation are unlikely to play a major role in cerebellar adaptations to opioid withdrawal. Although the cerebellum is not traditionally considered a major structure in addiction, the emerging evidence suggests that opioids affect its connectivity with key regions, such as the prefrontal cortex, the amygdala, and the striatum. Furthermore, some studies have shown that the cerebellum exhibits increased activity in response to opioid-related cues, suggesting a potential role in the craving and relapse processes. These findings highlight the need for further investigation into the cerebellum’s involvement in addiction-related neuroadaptations [69].

## 4. Materials and Methods

### 4.1. Materials

Morphine sulfate was obtained from Saneca Pharmaceuticals, Ltd. (Hlohovec, Slovakia). Some basic chemicals, including Tris, sucrose, glycine, and Tween 20, were purchased from Serva (Heidelberg, Germany). All other chemicals were obtained from Sigma-Aldrich (St. Louis, MO, USA).

### 4.2. Morphine Treatment

**Figure 5:**
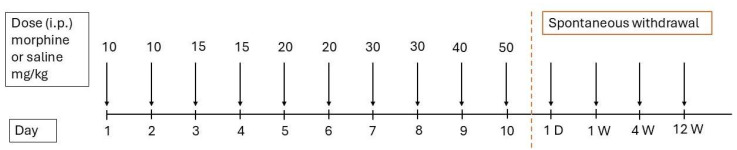
Timeline of morphine administration and spontaneous withdrawal.

Male Wistar rats (weighing approximately 300 g, 8 weeks old) were house under standard laboratory conditions (20–24 °C, 12/12-h light/dark cycle) with ad libitum access to chow diet and water. The animals were divided into four groups, each consisting of five rats. The rats in the morphine-treated groups received increasing doses of morphine sulfate (10–50 mg/kg) dissolved in 0.9% NaCl, administered intraperitoneally over 10 days: 10 mg/kg on days 1 and 2, 15 mg/kg on days 3 and 4, 20 mg/kg on days 5 and 6, 30 mg/kg on days 7 and 8, 40 mg/kg on day 9, and 50 mg/kg on day 10. The control groups received equivalent volumes of 0.9% NaCl via intraperitoneal injection. Following treatment, the animals underwent different withdrawal periods (1 day, 1 week, 4 weeks, or 12 weeks). At designated time points, the rats from both the morphine-treated and control groups were sacrificed by cervical dislocation. Specifically, the first group was euthanized 24 h after the last dose (1D), while the remaining groups were sacrificed at 1 week (1W), 4 weeks (4W), and 12 weeks (12W) post-morphine/saline administration, according to the experimental timeline (Figure 5). The brain regions of interest—the prefrontal cortex, the hippocampus, the striatum, and the cerebellum—were dissected, snap-frozen in liquid nitrogen, and stored at −80 °C until further analysis.

### 4.3. Sample Preparation

Total RNA and protein fractions were extracted from the brain tissue using TRIzol^®^ reagent (Invitrogen^TM^, Waltham, MA, USA) [70]. Samples were collected from four brain regions—the prefrontal cortex, the hippocampus, the striatum, and the cerebellum—of the rats at various withdrawal time points, as well as from the corresponding control group. Approximately 100 mg of brain tissue was placed into bead-containing tubes with 1 mL of TRIzol^®^ reagent. The tissue was homogenized using a BEADBUG™3 Microtube Homogenizer (Benchmark, Scientific, Sayreville, NJ, USA) at 300 rpm for 45 s. The homogenates were then incubated at room temperature for 5 min. Next, 200 μL of chloroform was added per 1 mL of TRIzol^®^ reagent, followed by thorough mixing and incubation at room temperature for 2–3 min. The samples were then centrifuged at 12,000× *g* for 15 min at 4 °C using a Hettich^®^ MIKRO 200/200R centrifuge (Andreas Hettich GmbH & Co. KG, Cuttlingen, Germany). Following centrifugation, the samples separated into three distinct phases: the upper aqueous phase containing RNA, the interphase containing DNA, and the lower phenol-chloroform phase containing proteins.

### 4.4. RNA and Protein Isolation

The upper aqueous phase containing RNA was carefully transferred into a sterile 1.5 mL tube using a pipette. To precipitate RNA, 500 μL of isopropanol was added to the aqueous phase, followed by thorough mixing and incubation on ice for 10 min. After incubation, the sample was centrifuged at 12,000× *g* for 10 min at 4 °C using a Hettich^®^ MIKRO 200/200R centrifuge. The supernatant was then removed, and 1 mL of 75% ethanol was added to the pellet. The sample was briefly vortexed and centrifuged at 7500× *g* for 5 min at 4 °C. The supernatant was discarded, and another 1 mL of 75% ethanol was added to the pellet. This washing step was repeated once more to enhance RNA purification. After the final ethanol wash, the pellet was left to air-dry in an open tube for 5–10 min under a fume hood. Once dried, the RNA pellet was resuspended in 35 μL of RNase-free water and incubated for 10 min at 60 °C on a Techne^®^ Dri-Block^®^ heater (Bibby Scientific Ltd., Staffordshire, UK). The concentration and purity of the RNA samples were measured using a NanoDrop™ One Microvolume UV-Vis Spectrophotometer (Thermo FisherScientific, Waltham, MA, USA). The A260/A280 ratio of pure RNA should be approximately 2.0, which was consistent with the values obtained for the isolated RNA samples. The purified RNA was subsequently stored at −80 °C for future use. To the phenol-ethanol phase, 1.5 mL of isopropanol was added, followed by incubation at room temperature for 10 min. The samples were then centrifuged at 1200× *g* for 10 min at 4 °C, resulting in the formation of a pellet at the bottom of the tube. The supernatant was carefully removed. The pellet was washed with 2 mL of washing buffer (300 mM guanidine hydrochloride and 95% ethanol) and incubated at room temperature for 20 min with constant mixing. Subsequently, the samples were centrifuged at 7500× *g* for 5 min at 4 °C. The supernatant was discarded, and the washing step was repeated twice to enhance protein precipitation. Next, 2 mL of 100% ethanol was added to the pellet, followed by incubation at room temperature for 20 min with continuous mixing. After incubation, the samples were centrifuged at 7500× *g* for 5 min at 4 °C. The supernatant was carefully removed, and the pellet was left to air-dry in an open tube for 5–10 min. The dried pellet was resuspended in 200 μL of buffer (1 M Tris base, 35 mM SDS, 8 M urea; pH 8.0) supplemented with cOmplete™ Protease Inhibitor Cocktail (Roche Diagnostics GmbH, Mannheim, Germany) and PhosSTOP™ Phosphatase Inhibitor Cocktail (Sigma-Aldrich, Darmstadt, Germany)). The resuspended pellet was then sonicated using a Bandelin SONOPULS ultrasonic sonicator (Bandelin electronic GmbH & Co. KG, Berlin, Germany) in five cycles of 15 s each at 50% amplitude. The samples were subsequently centrifuged at 10,000× *g* for 10 min at 4 °C. The resulting supernatant containing the extracted proteins was carefully collected and transferred into new sterile 1.5 mL tubes. The protein samples were flash-frozen in liquid nitrogen and stored at −80 °C. Protein concentrations were determined using the bicinchoninic acid (BCA) assay method [71].

### 4.5. Assessment of m^6^A RNA Modification

The m^6^A RNA methylation levels were quantified using the EpiQuik™ m^6^A RNA Methylation Quantification Kit (Epigentek, New York, NY, USA) following the manufacturer’s protocol. For each sample, 200 ng of total RNA was used. The assay was performed according to the recommended procedure, including incubation with specific reagents and subsequent absorbance measurement at 450 nm using a Biotek Synergy HT Microplate Reader (BioTek Instruments, Winooski, VT, USA).

### 4.6. Western Blotting

Isolated proteins were prepared for electrophoresis at a final concentration of 1 μg/μL in a total volume of 100 μL, with one-quarter of the volume consisting of Laemmli buffer. The samples were thoroughly mixed, incubated in a Techne^®^ Dri-Block^®^ heater at 95 °C for 3 min, and then loaded onto standard 10% polyacrylamide gels following previously established protocols [72,73]. Electrophoresis was conducted at 200 V for 45 min. Following electrophoresis, the proteins were transferred to a nitrocellulose membrane (Protran BA85, GE Healthcare, Chalfont St Giles, UK) at 100 V for 90 min. The membranes were then blocked with 5% nonfat dry milk in TBS buffer (10 mM Tris, 150 mM NaCl, pH 8.0) for 40 min before overnight incubation at 4 °C with primary antibodies specific to the following m^6^A-related proteins: METTL3 (rabbit monoclonal, 70 kDa, Cell Signaling Technology, catalog number 96391; dilution 1:1000), FTO (mouse monoclonal, 58 kDa, Abcam, catalog number ab92821, dilution 1:1000), ALKBH5 (rabbit polyclonal, 42 kDa, Proteintech, catalog number 16837-1-AP; dilution 1:1000), and YTHDF1 (rabbit polyclonal, 60 kDa, Proteintech, catalog number 17479-1-AP; dilution 1:1000). The next day, the membranes were washed three times for 10 min in TBS-T buffer (TBS with 0.3% Tween 20), followed by incubation with horseradish peroxidase (HRP)-conjugated secondary anti-mouse (Amersham, UK, catalog number NA931; dilution 1:20,000) or anti-rabbit IgG antibodies (Amersham, UK, catalog number NA939; dilution 1:40,000) for 1 h at room temperature. After another three 10 min washes in TBS-T, the protein bands were visualized using the enhanced chemiluminescence (ECL) technique with SuperSignal^TM^ West Dura substrate (Pierce Biotechnology, Rockford, IL, USA). To verify uniform sample loading, nitrocellulose membranes were stained with Ponceau S dye. Western blot signals were quantified using ImageLab software, version 6.1.0 (Bio-Rad Laboratories, Hercules, CA, USA), with the band intensities normalized to the total protein content (Ponceau staining).

### 4.7. Statistical Analysis

Statistical analysis was performed using GraphPad Prism software, version 10.0 (GraphPad Software, La Jolla, CA, USA). The outliers were identified and removed using Grubbs’ test (GraphPad QuickCalcs, GraphPad Software). To compare the differences between the groups, two-way analysis of variance (two-way ANOVA) was performed, followed by Bonferroni’s post hoc test for multiple comparisons. Quantitative variables are presented as the mean ± standard error of the mean (SEM). Statistical significance was set at *p* < 0.05. Each experimental group consisted of five biological replicates (animals). The normalization of Western blot results to total protein loading was performed using Ponceau S staining (SERVA Electrophoresis GmbH, Heidelberg, Germany), which served as a control for uniform protein loading. The signal intensity of the detected proteins was related to that of an internal standard, which consisted of a rat brain tissue sample processed using the same protein isolation method as the test samples. The signal intensity of the target protein in this internal standard was set to 100%, and all other values are expressed as percentages relative to this reference. A single value from each sample was used for statistical analysis. The percentage of m^6^A was measured using the EpiQuik™ m^6^A RNA Methylation Quantification Kit (Epigentek, New York, NY, USA), a specific ELISA-based assay. The percentage of m^6^A (%) was calculated using the following formula:

$$m^{6}A\%=\frac{(Sample\;OD\;-\;NC\;OD)\;÷\;S}{(PC\;OD\;-\;NC\;OD)\;÷\;P}\times 100\%$$

In this formula, Sample OD represents the optical density of the unknown sample, NC OD represents the optical density of the negative control, and PC OD represents the optical density of the positive control. The variable S corresponds to the initial RNA amount (in ng), while P represents the initial amount of the positive control (in ng). For statistical analysis, a single value from each sample was used. The results are presented as the mean ± SEM based on five biological replicates (n = 5).

## 5. Conclusions

Our study demonstrates that m^6^A modification and its regulatory enzymes undergo region-specific and time-dependent alterations in response to chronic morphine exposure and subsequent withdrawal. While the m^6^A and METTL3 levels decreased in both the prefrontal cortex and the striatum, these changes emerged later in the striatum, becoming evident at 4 weeks of withdrawal. In contrast, m^6^A modification remained stable in the hippocampus and the cerebellum, with no significant changes observed in YTHDF1 expression. This suggests that while some brain regions undergo rapid epitranscriptomic shifts, others may exhibit delayed or minimal responses to opioid withdrawal. These findings highlight that opioid withdrawal induces transient, brain-region-specific epitranscriptomic changes, which may play a role in neuroadaptive processes associated with addiction and withdrawal.

## Data Availability

Data are contained within this article and the Supplementary Materials.

## Supplementary Materials

The following supporting information can be downloaded at: https://www.mdpi.com/article/10.3390/ijms26094371/s1.

## Abbreviations

The following abbreviations are used in this manuscript:

C	control
FTO	fat mass and obesity-associated gene
METTL3	methyltransferase-like 3
METTL14	methyltransferase-like 14
M_W_	morphine withdrawal
m⁶A	N6-methyladenosine
SAM	S-adenosyl methionine
TBS	Tris-buffered saline
1D	1 day
1W	1 week
4W	4 weeks
12W	12 weeks
