# Supplementary material for: An Investigation of the RNA Modification m6A and Its Regulatory Enzymes in Rat Brains Affected by Chronic Morphine Treatment and Withdrawal
Source: Int J Mol Sci. 2025 May 4;26(9):4371. doi: 10.3390/ijms26094371 (PMC12072463; doi:10.3390/ijms26094371)

Supplementary Material

Original images of immunoblots and Ponceau S staining. Proteins from samples of selected brain regions (prefrontal cortex, hippocampus, striatum, and cerebellum) of rats administered morphine for 10 days, followed by different periods of abstinence (1 day, 1 week, 4 weeks, and 12 weeks), were resolved by SDS electrophoresis and immunoblotted using antibodies against the methyltransferase METTL3, demethylases ALKBH5 and FTO, and the reader protein YTHDF1.

I. YTHDF1

a. Prefrontal cortex

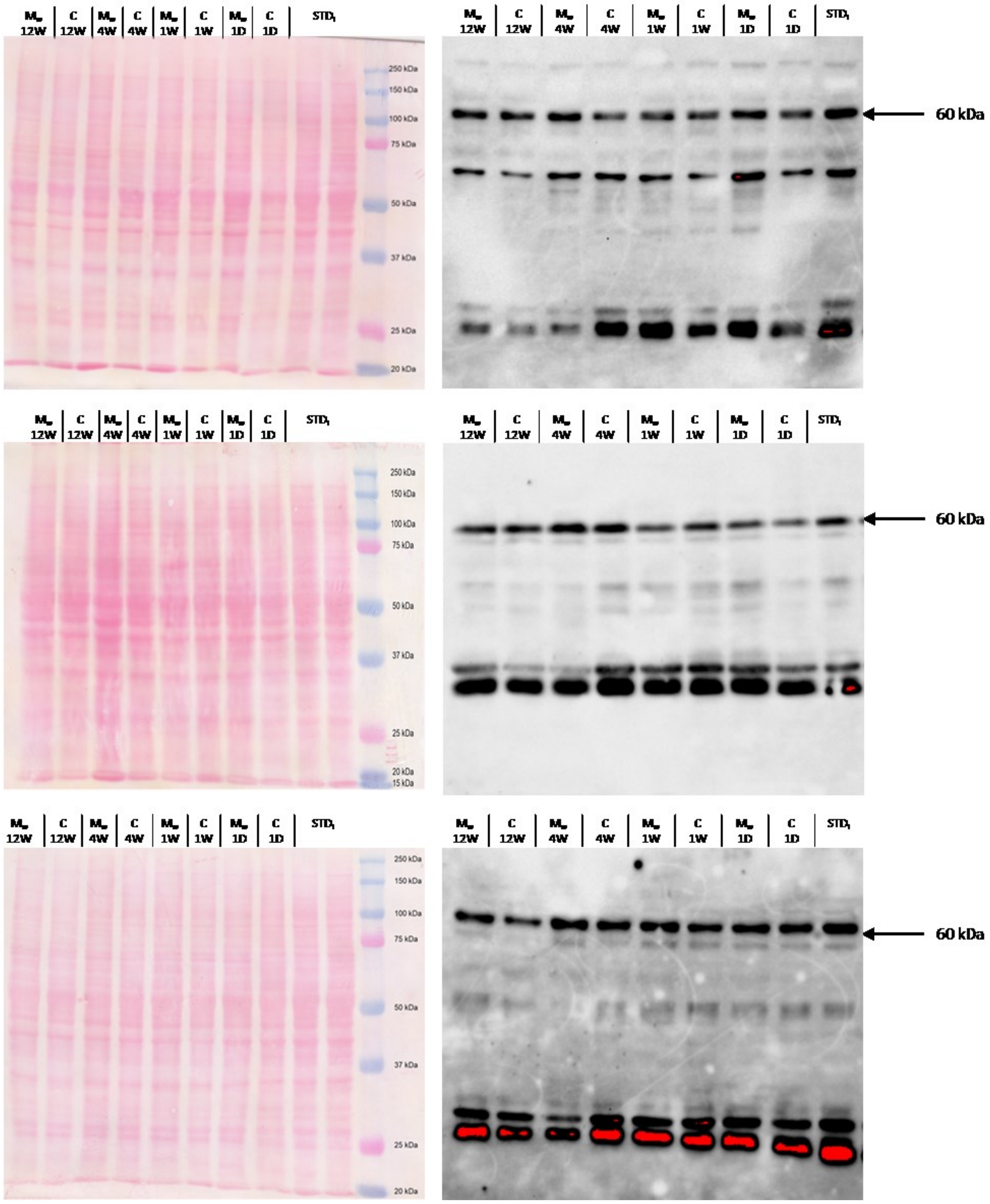

b. Hippocampus

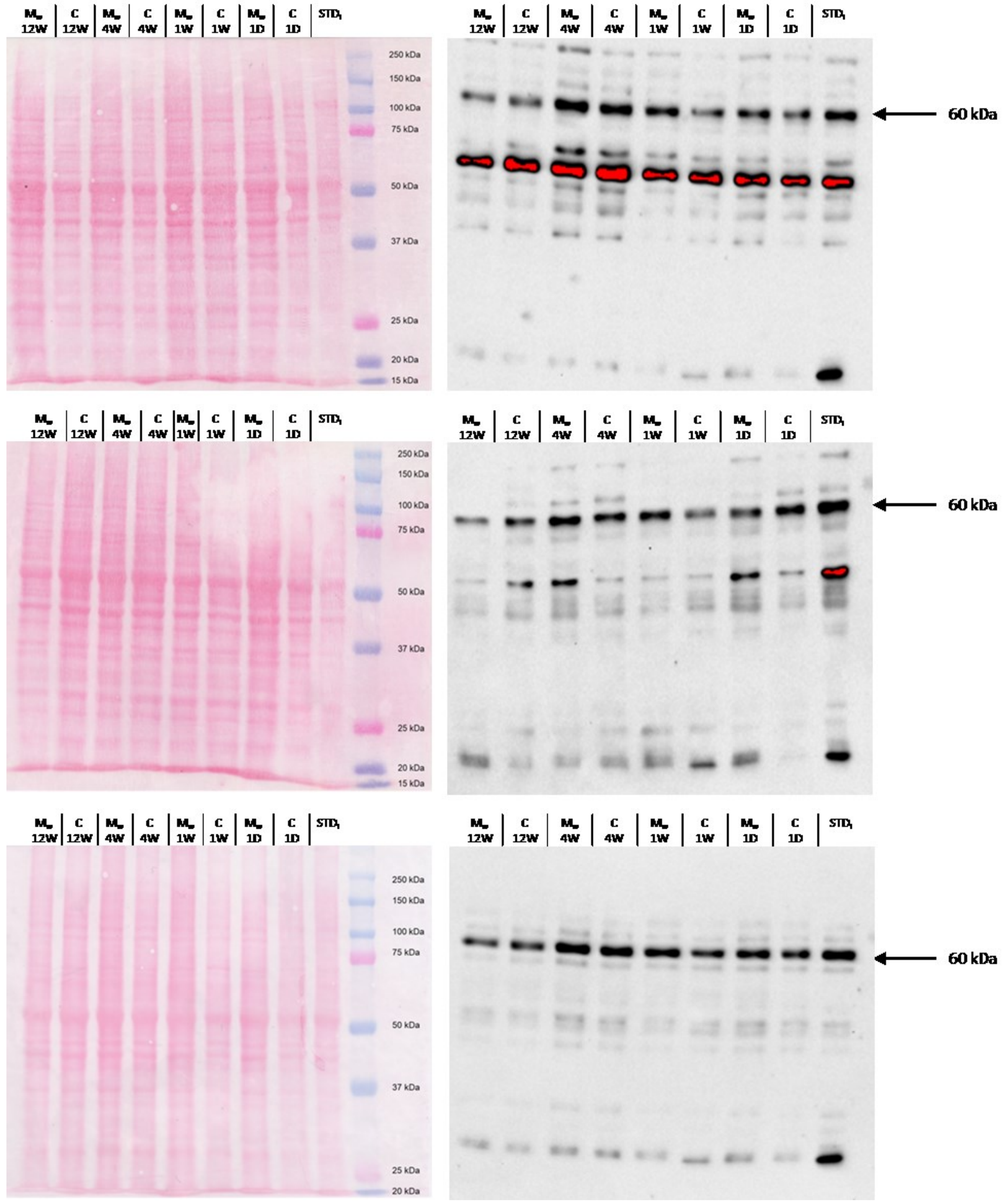

c. Striatum

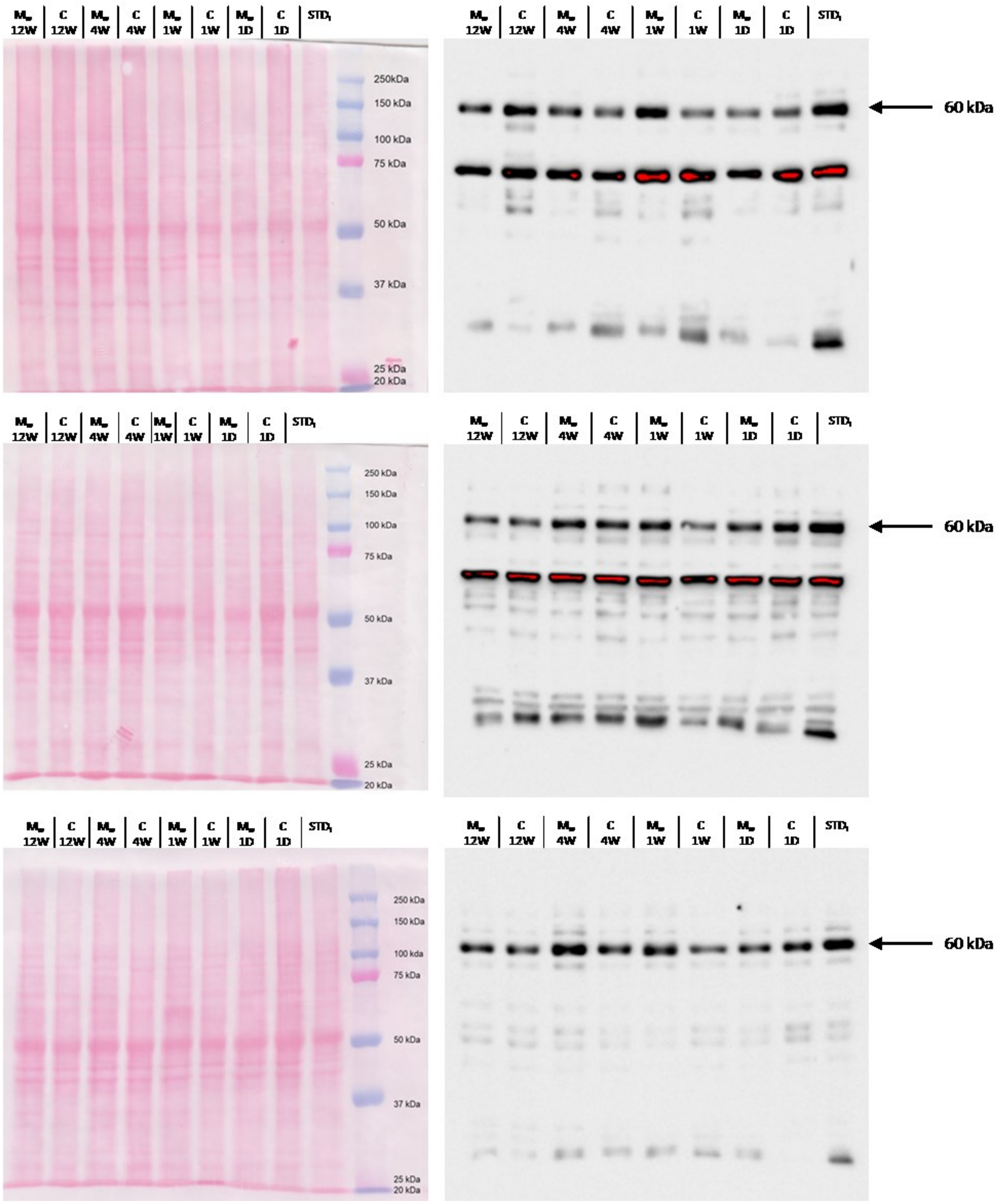

d. Cerebellum

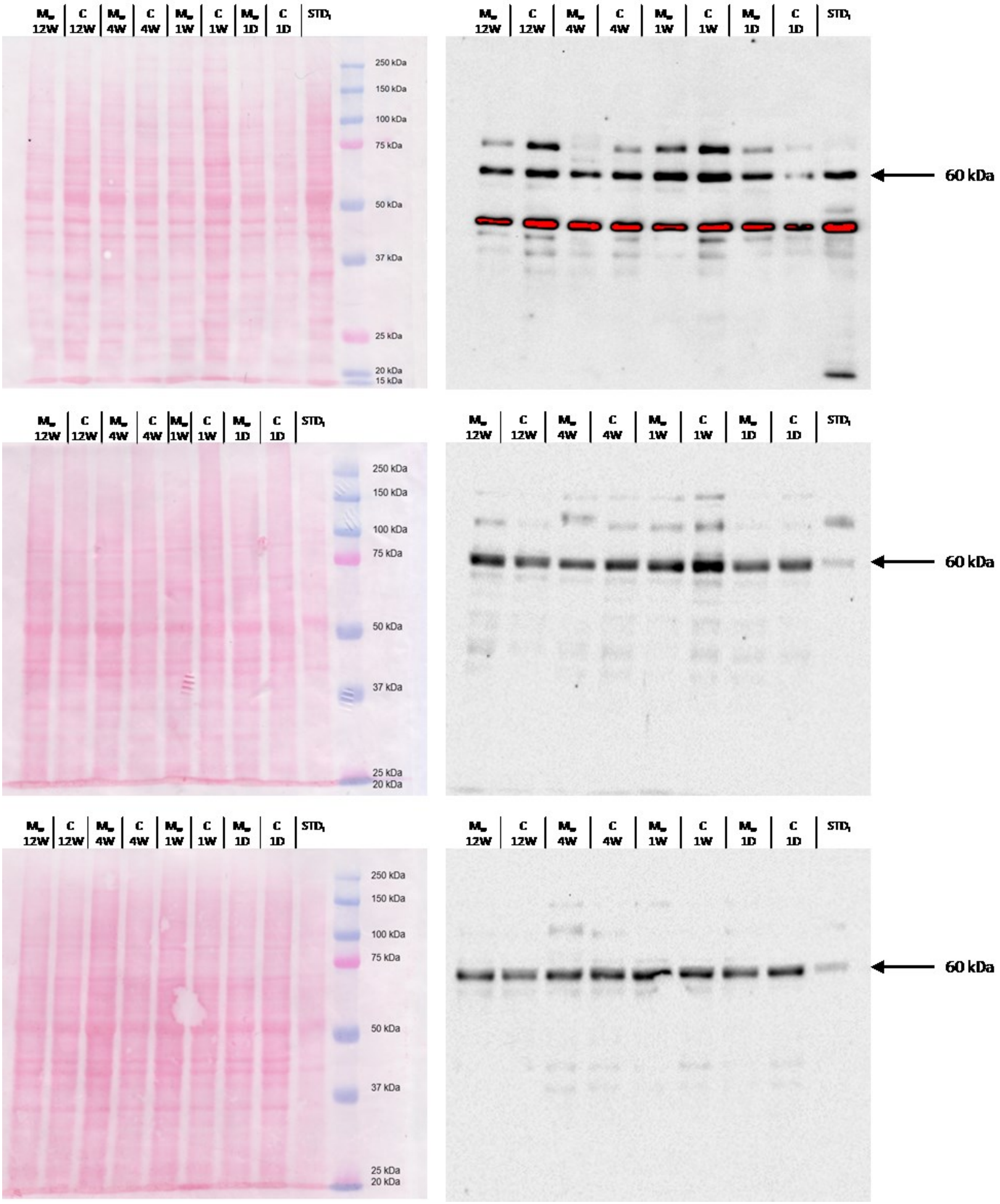

II. ALKBH5

a. Prefrontal cortex

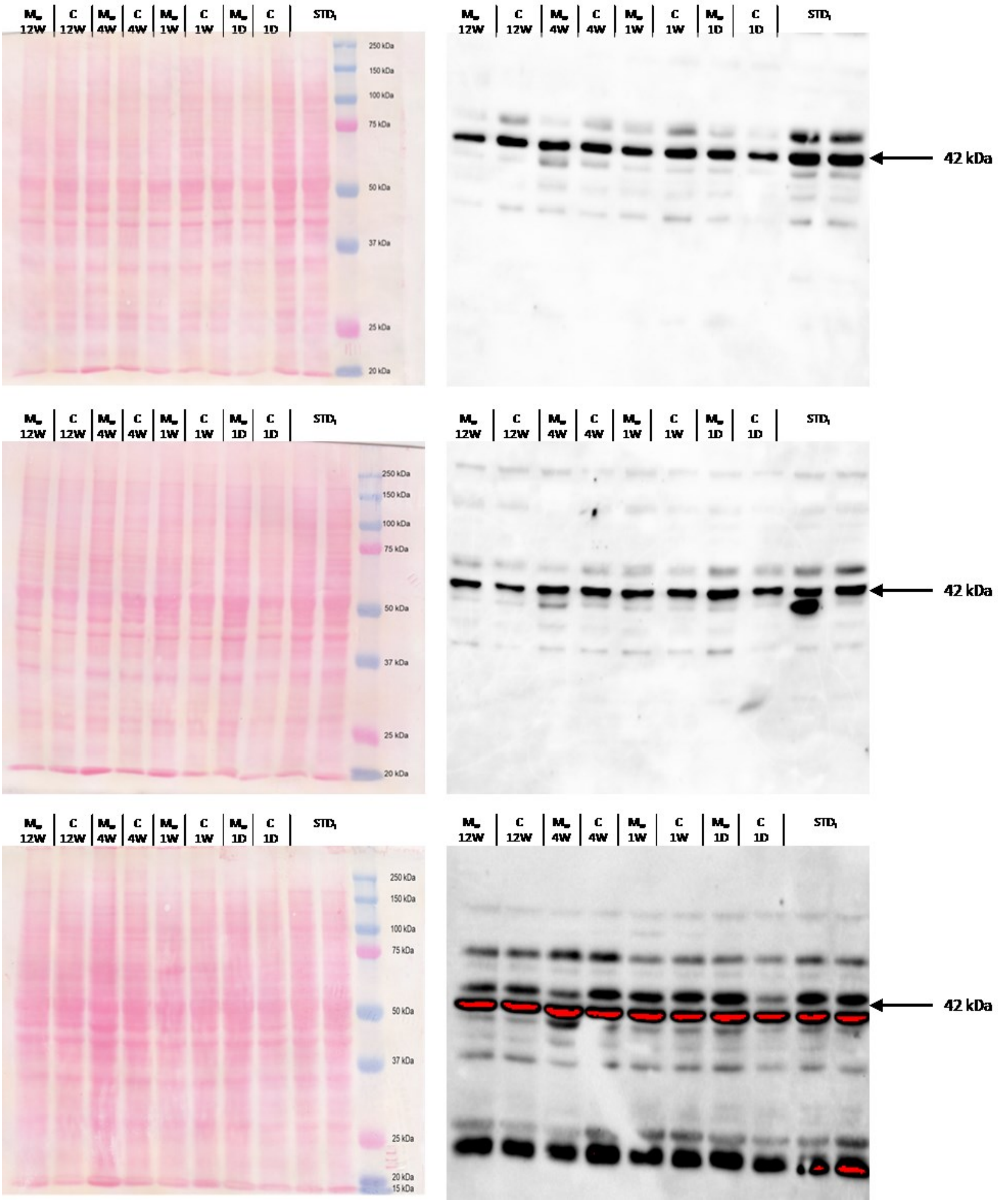

b. Hippocamous

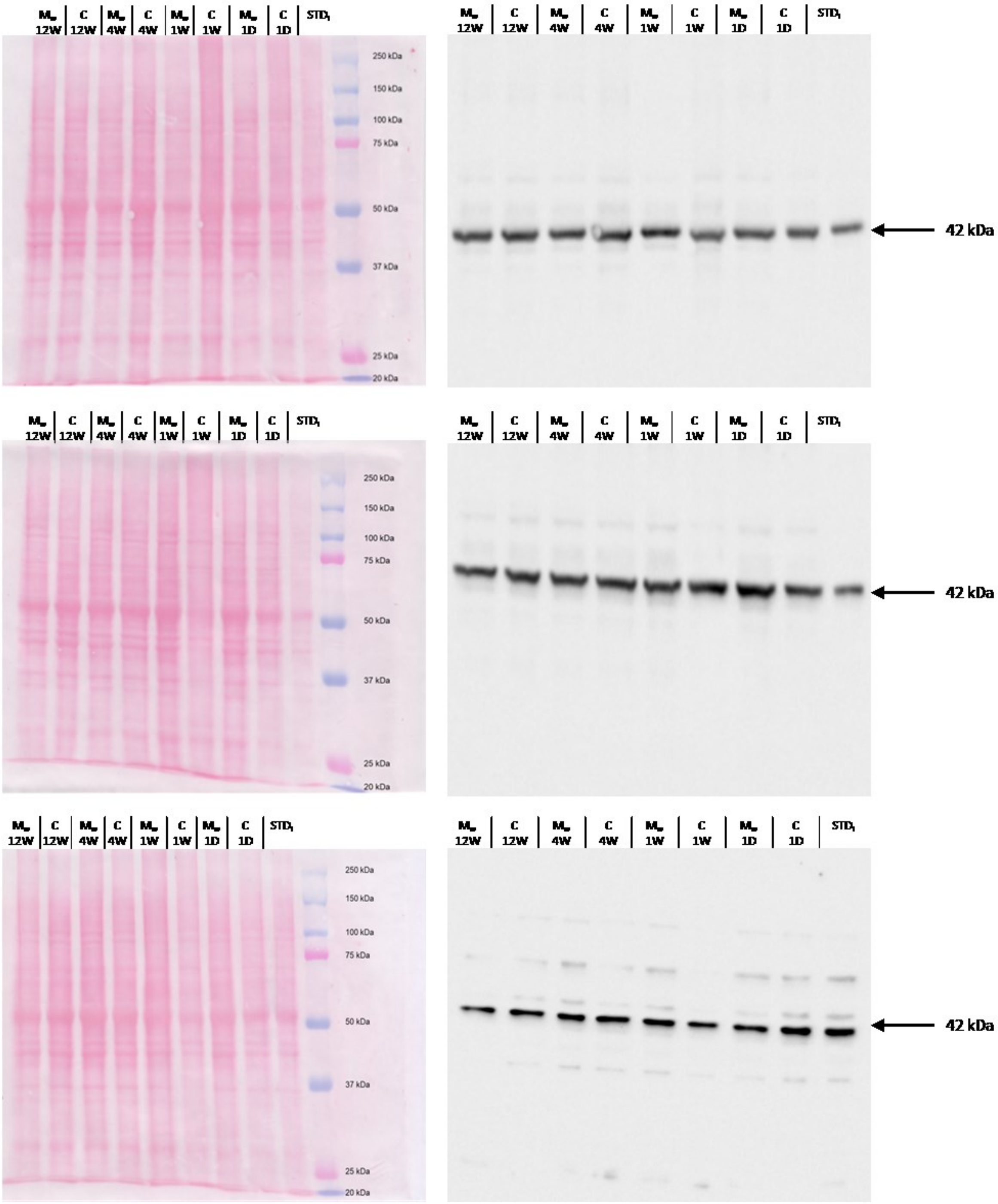
M 12W
C 12W
M 4W
C 4W
M 1W
C 1W
M 1D
C 1D
STD
250 kDa
150 kDa
100 kDa
75 kDa
50 kDa
37 kDa
25 kDa
20 kDa
42 kDa

c. **Striatum**

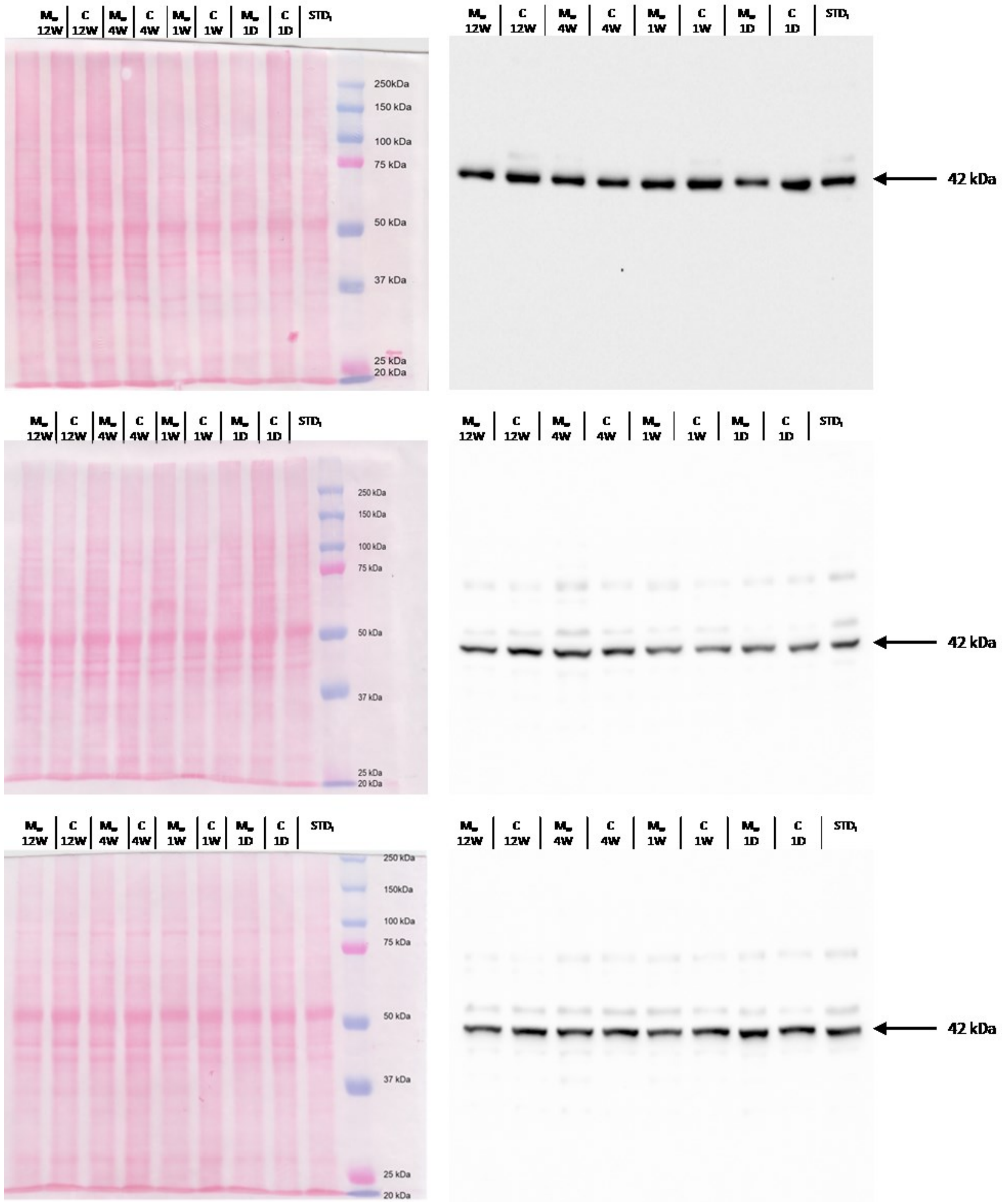
M 12W | C 12W | M 4W | C 4W | M 1W | C 1W | M 1D | C 1D | STD
250kDa
150 kDa
100 kDa
75 kDa
50 kDa
37 kDa
25 kDa
20 kDa
M 12W | C 12W | M 4W | C 4W | M 1W | C 1W | M 1D | C 1D | STD
42 kDa
M 12W | C 12W | M 4W | C 4W | M 1W | C 1W | M 1D | C 1D | STD
250 kDa
150 kDa
100 kDa
75 kDa
50 kDa
37 kDa
25 kDa
20 kDa
M 12W | C 12W | M 4W | C 4W | M 1W | C 1W | M 1D | C 1D | STD
42 kDa
M 12W | C 12W | M 4W | C 4W | M 1W | C 1W | M 1D | C 1D | STD
250 kDa
150kDa
100 kDa
75 kDa
50 kDa
37 kDa
25 kDa
20 kDa
M 12W | C 12W | M 4W | C 4W | M 1W | C 1W | M 1D | C 1D | STD
42 kDa

d. Cerebellum

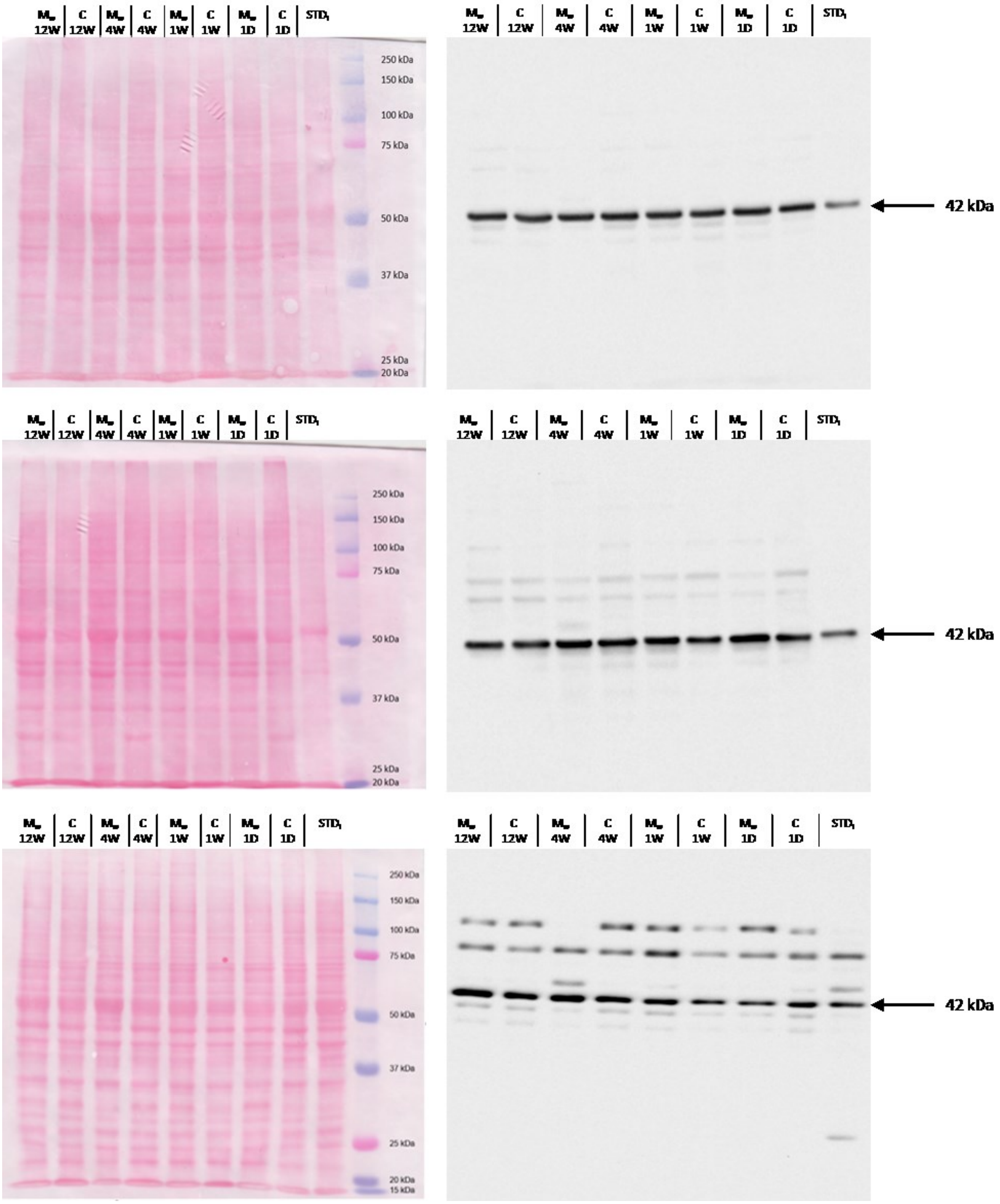

III. FTO

a. Prefrontal cortex

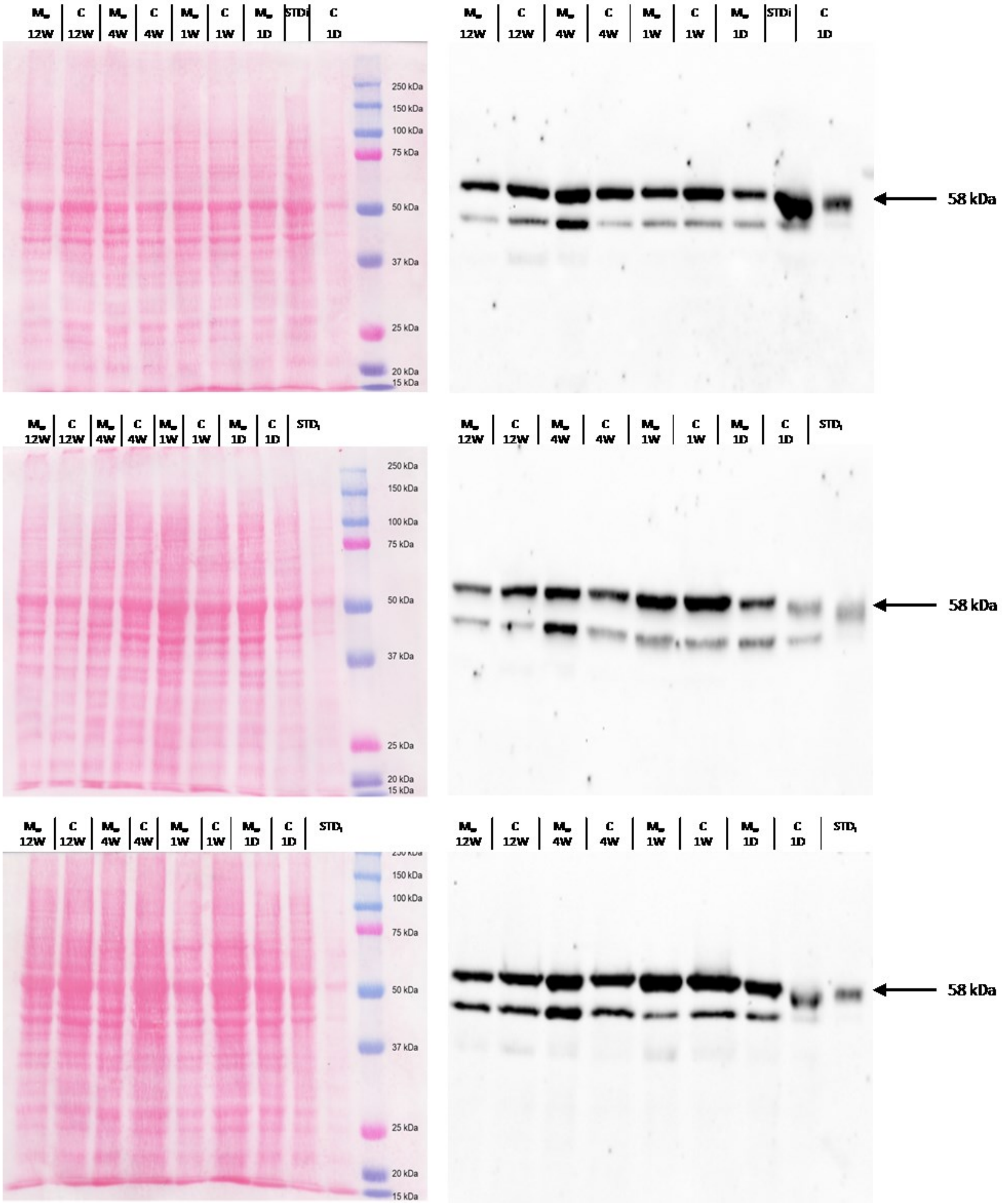

b. Hippocampus

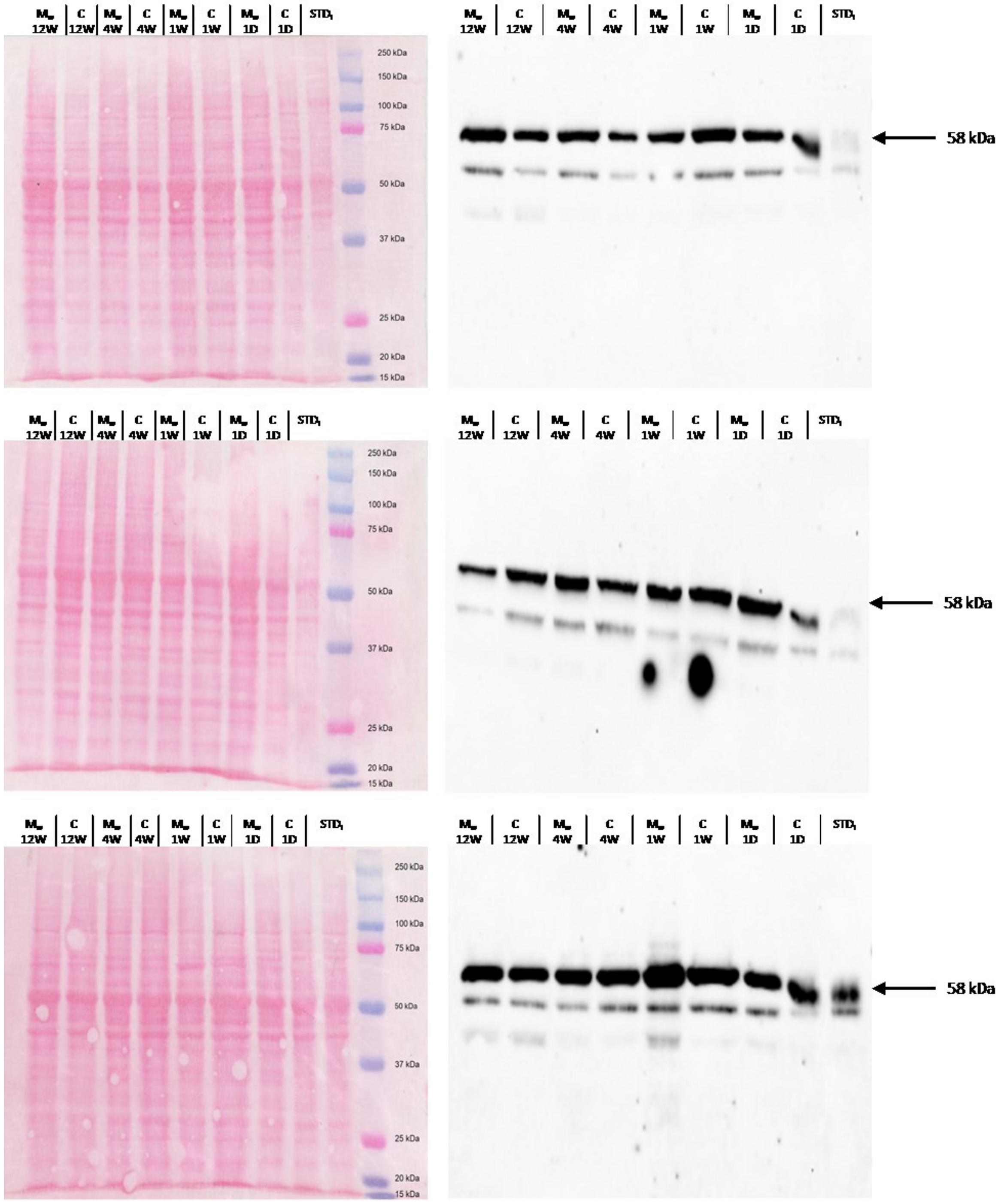

c. **Striatum**

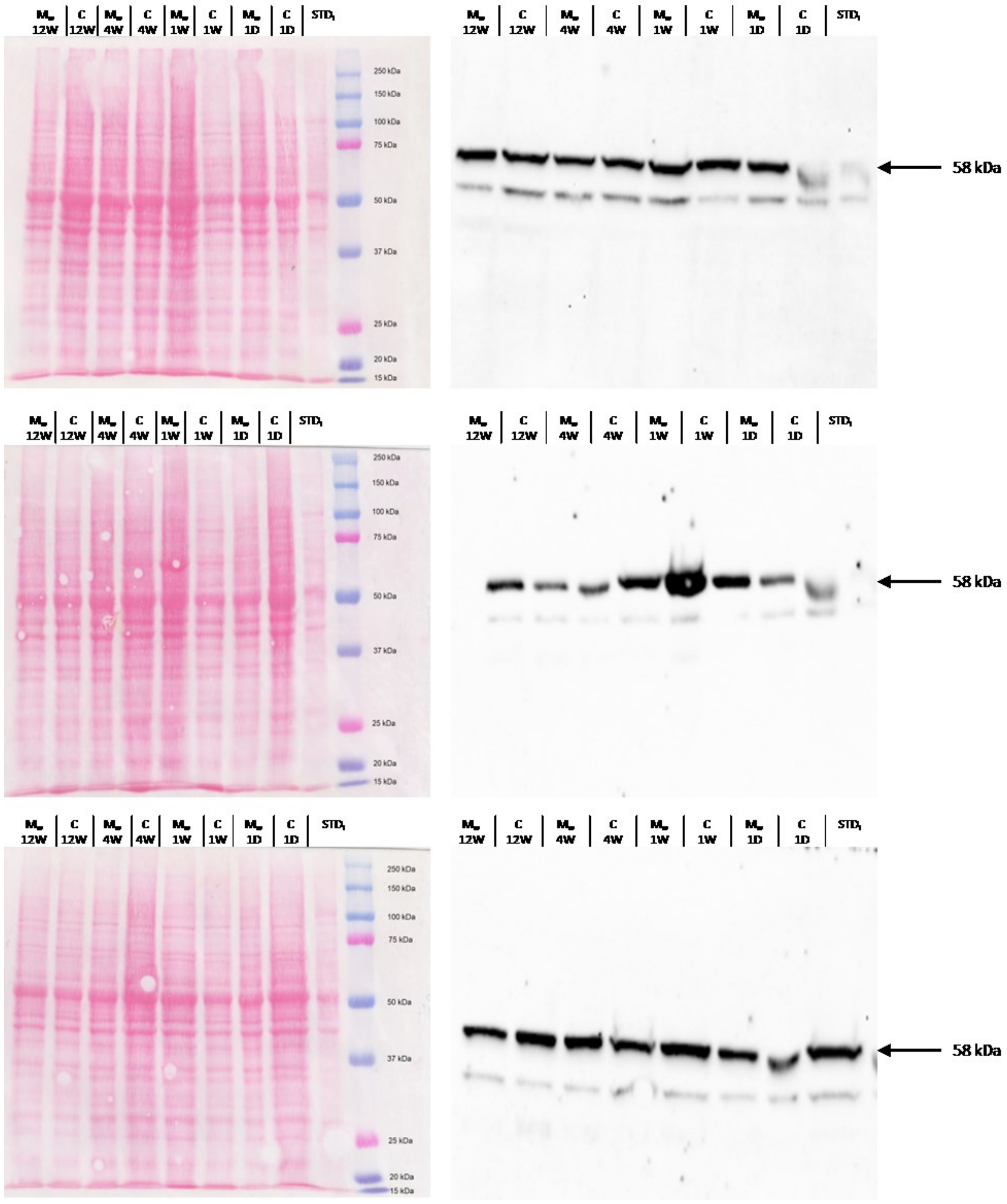

M 12W
C 12W
M 4W
C 4W
M 1W
C 1W
M 1D
C 1D
STD
250 kDa
150 kDa
100 kDa
75 kDa
50 kDa
37 kDa
25 kDa
20 kDa
15 kDa
58 kDa

d. Cerebellum

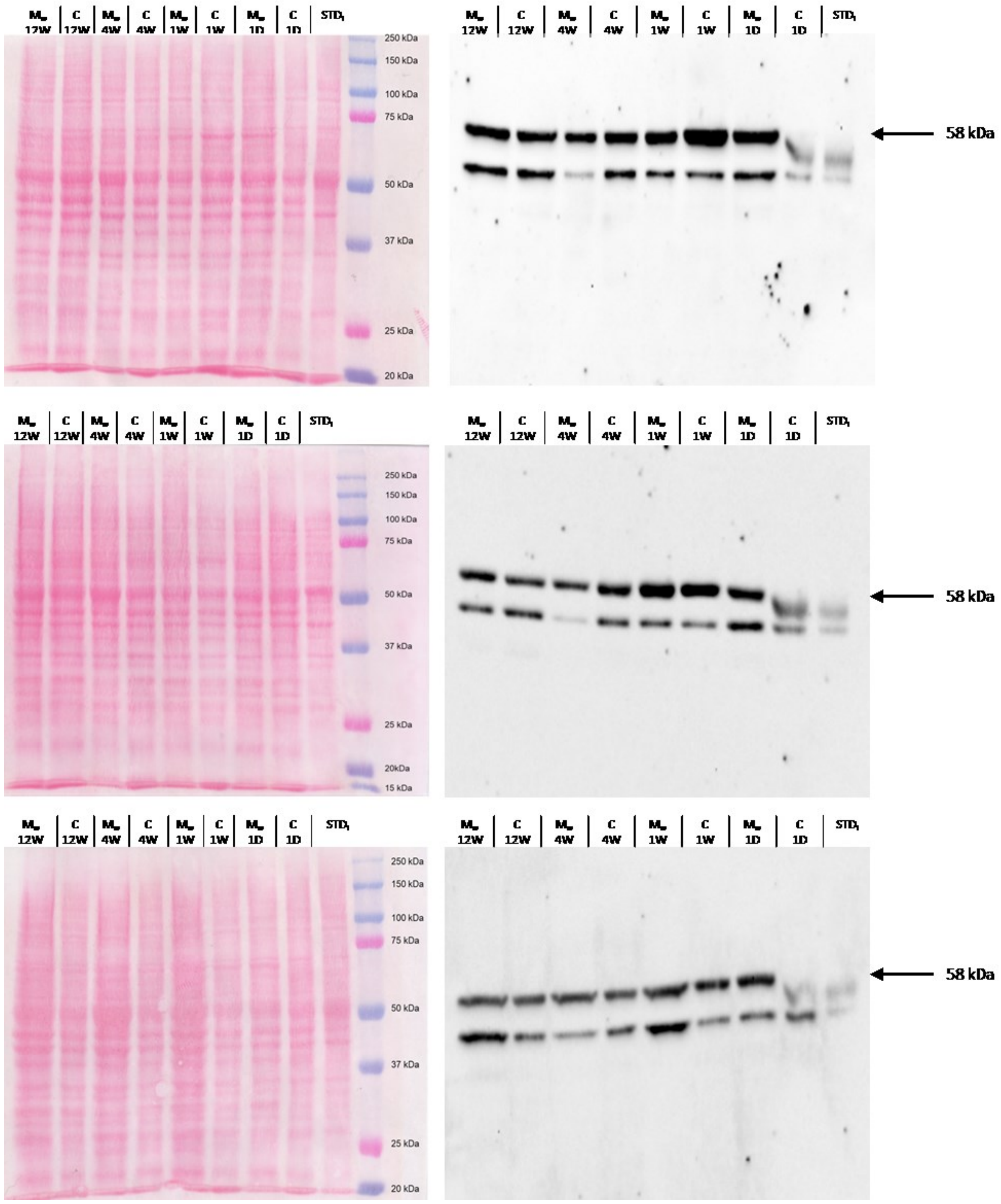

IV. METTL3

a. Prefrontal cortex

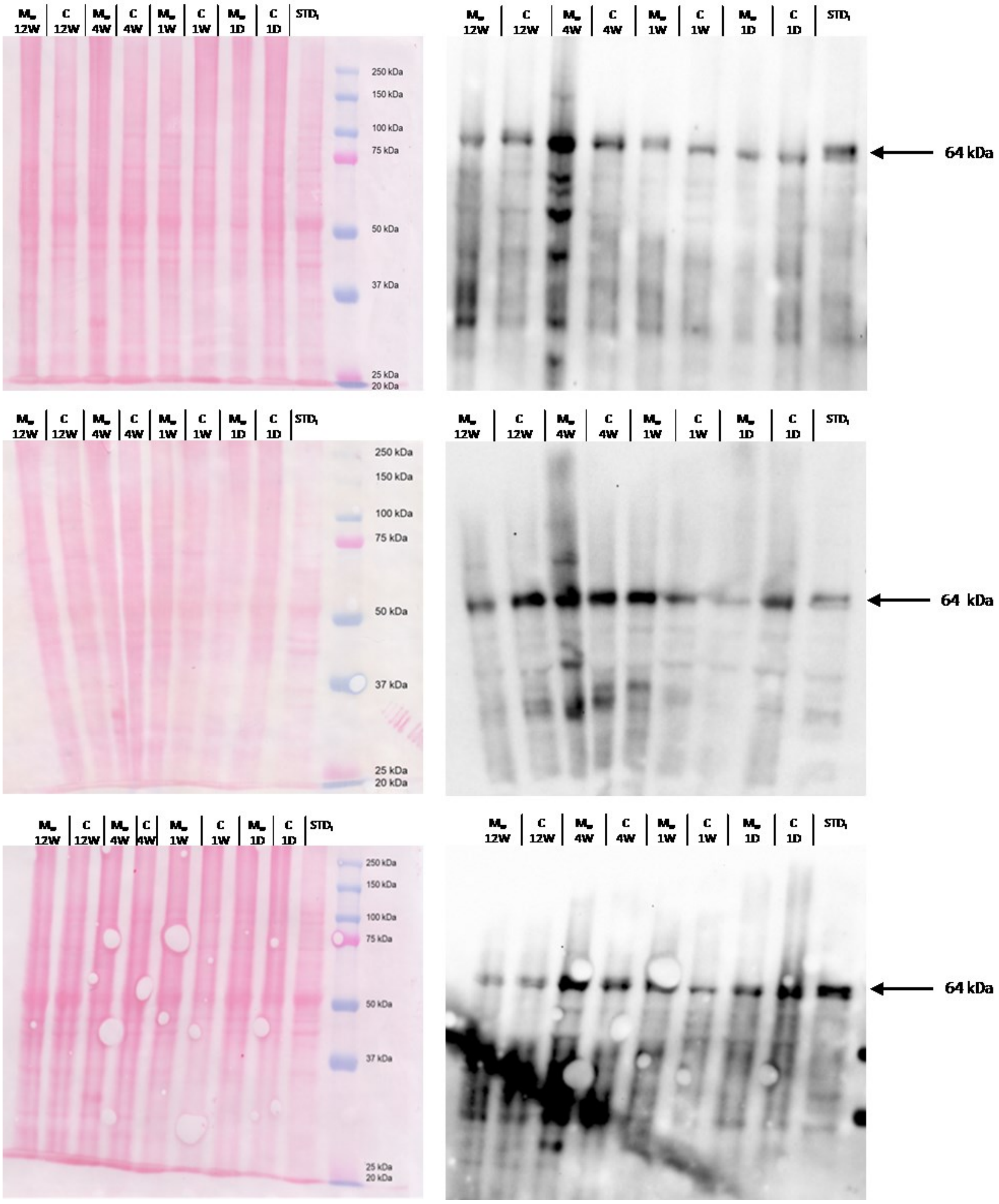

b. Hippocampus

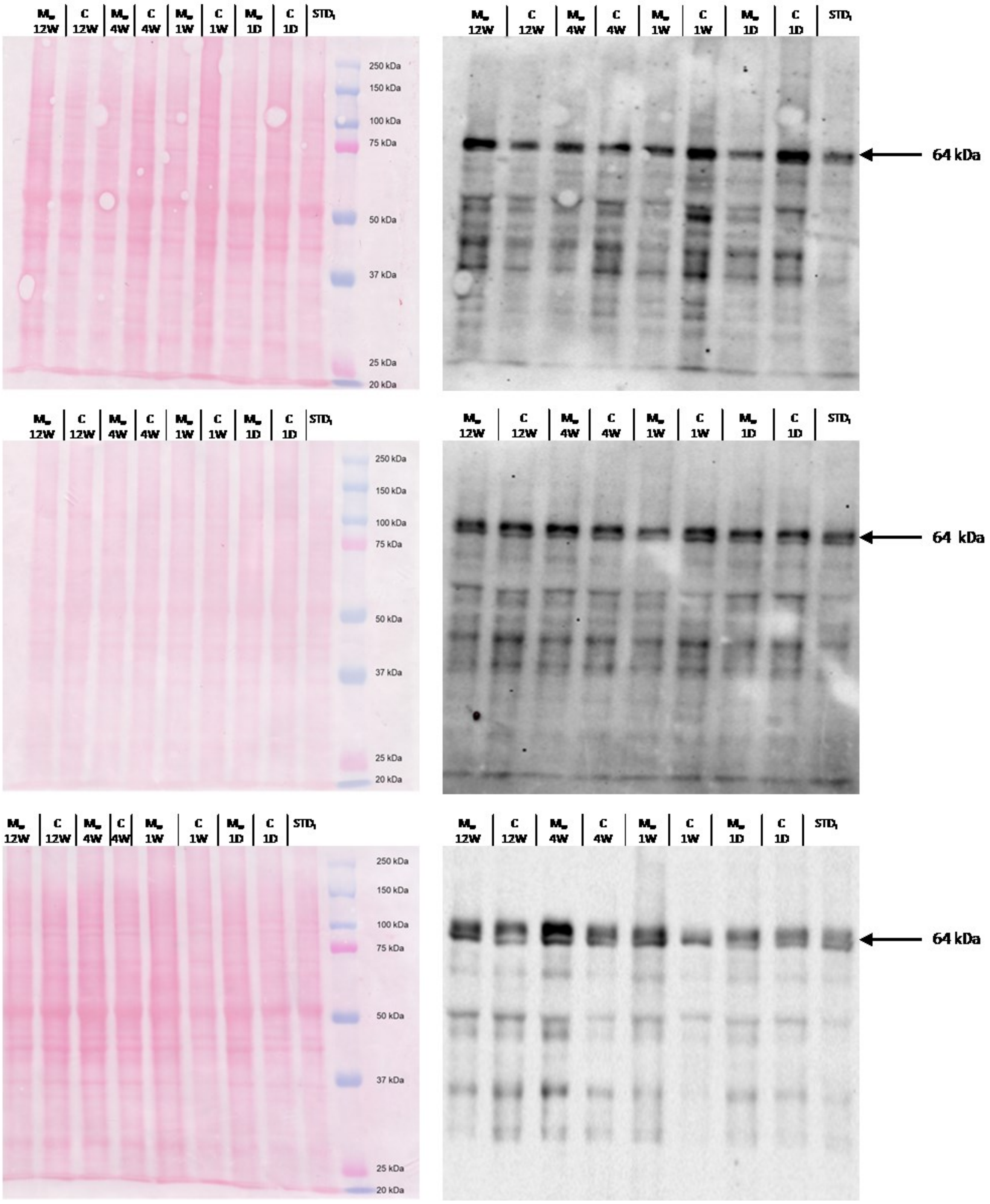

c. Striatum

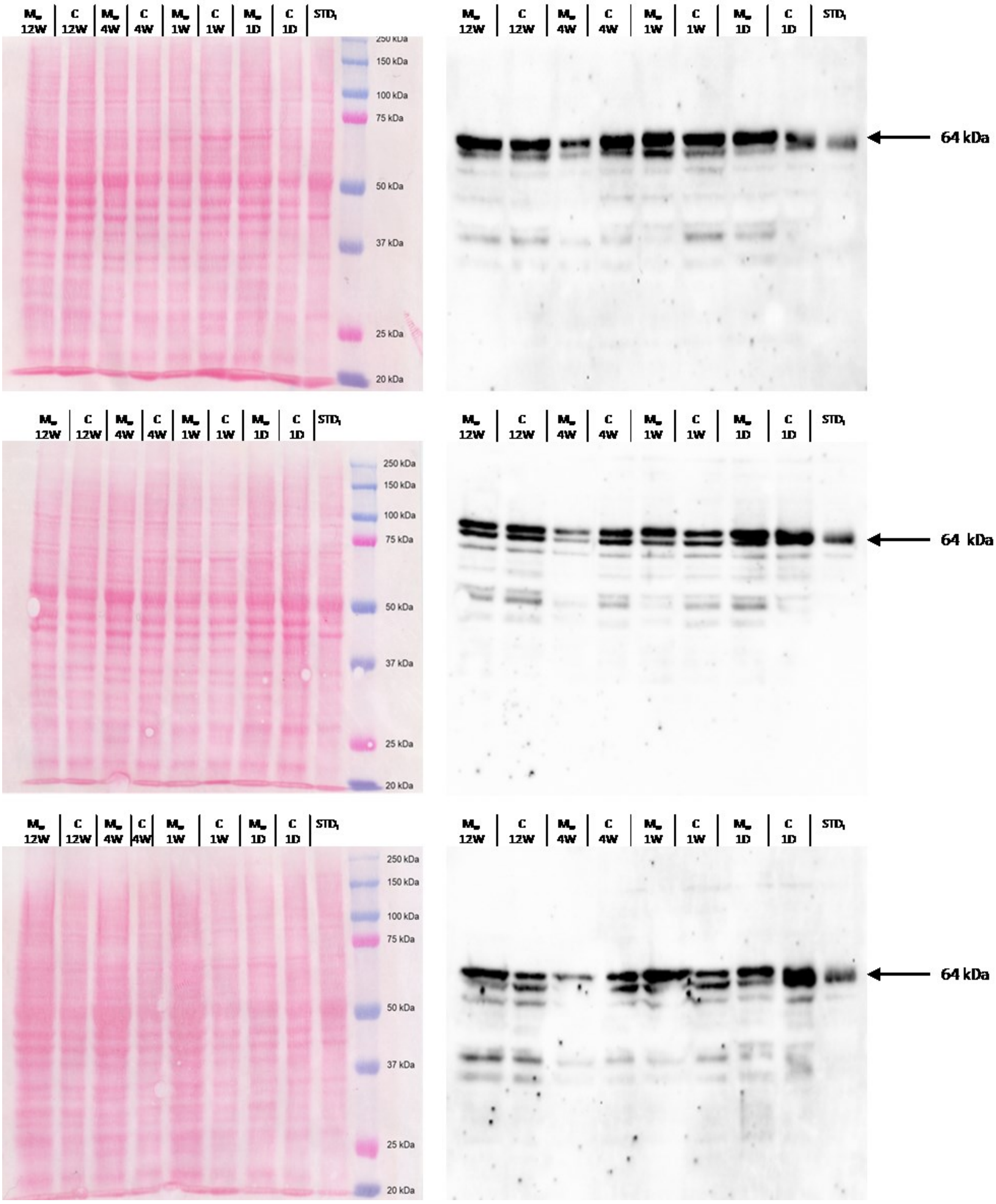
M 12W
C 12W
M 4W
C 4W
M 1W
C 1W
M 1D
C 1D
STD
250 kDa
150 kDa
100 kDa
75 kDa
50 kDa
37 kDa
25 kDa
20 kDa
64 kDa

d. Cerebellum

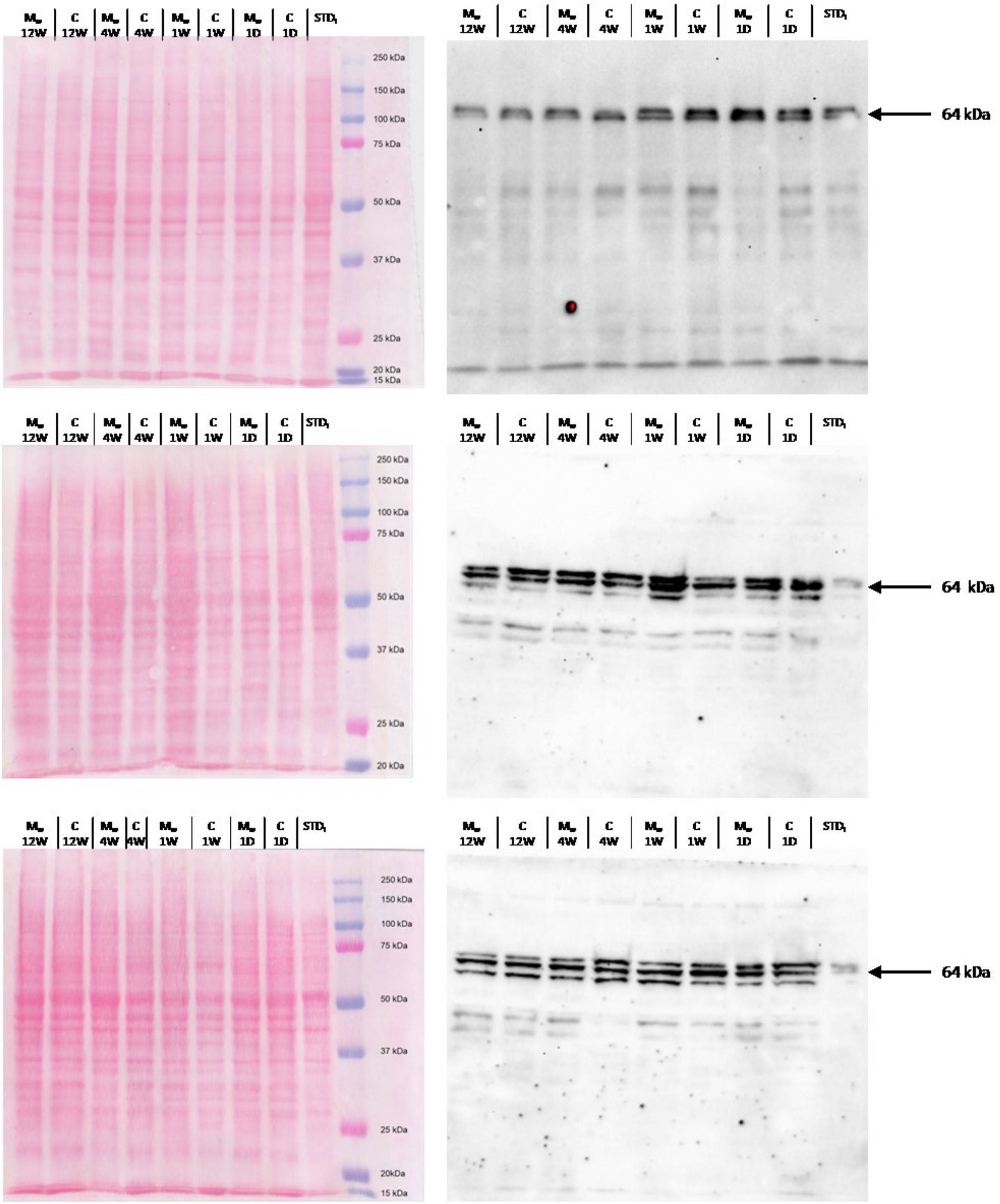

Supplement: Supplementary file 1 [file ijms-26-04371-s001.zip › ijms-3575080-supplementary.pdf]
